# The role of leukotriene inhibition using a 5-lipoxygenase (5-LO) inhibitor in a joint contracture model

**DOI:** 10.1186/s40634-023-00616-w

**Published:** 2023-06-21

**Authors:** Alexander D. Jeffs, Michael Boyd, Landon Larabee, Matthew Shelton, Alexander Bassil, Ross Taylor, David Berkoff

**Affiliations:** 1 Department of Orthopaedics, The University of North Carolina, Chapel Hill, NC USA; 2 Department of Family Medicine, The University of North Carolina, Chapel Hill, NC USA; 3 The University of North Carolina School of Medicine, Chapel Hill, NC USA; 4 Duke University School of Medicine, Durham, NC USA; 5 Wake Forest University School of Medicine, Winston Salem, NC USA

**Keywords:** Arthrofibrosis, Leukotriene, Contracture, Caffeic Acid, And 5-Lipoxygenase

## Abstract

**Purpose:**

Arthrofibrosis is a common inflammatory complication of joint trauma and surgery. 5lipoxygenase (5-LO) is a key enzyme involved in inflammation. Inhibition of 5-LO has been shown to reduce inflammation in heart and lung models but has not been examined in a joint contracture model.

**Methods:**

Twenty-six rats underwent joint contracture. Six rats served as non-surgical controls. A 5-LO inhibitor, caffeic acid (CA), suspended in 10% ethanol was orally administered to 14 rats and ethanol without CA to the remaining 12 rats daily for 21 days. Leukotriene B4 (LTB4) levels were measured, both systemically and locally. 5-LO levels in the posterior capsule were quantified by measuring the ratio of the length of the posterior capsule demonstrating 5-LO immunostaining to the total length of the capsule.

**Results:**

Joint contracture was successfully achieved in all rats who underwent manipulation. Levels of 5- LO measured in the posterior capsule were significantly increased in the animals who underwent surgery (56%/44–64) compared to the non-surgical control animals (7%/4–9). LTB4 levels were found to be significantly lower in the non-surgical control animals (107.79 ± 34.08 pg/ml) compared to all surgical animals (157.6 ± 55.3 pg/ml).

**Conclusion:**

Surgical intervention resulted in increased 5-LO activity of the synovial surface of the posterior capsule and increased LTB4 levels in the patellar tendon—fat pad. Oral administration of the 5LO inhibitor, CA, was ineffective at reducing systemic and local LTB4 levels and preventing knee joint contracture. Inhibiting 5-LO activity may still be effective in preventing arthrofibrosis and warrants further investigation.

## Background

Arthrofibrosis is a complication of joint trauma and surgical intervention [1]. An exaggerated inflammatory response leads to excessive production of fibrotic scar tissue, which causes the pain and joint stiffness seen in arthrofibrosis [2]. This sequela is well-known after total knee arthroplasty (TKA) and anterior cruciate ligament (ACL) reconstructions and can lead to patient dissatisfaction as well as costly revision surgeries [3, 4]. The frequency of these surgeries being performed and thus the risk of developing arthrofibrosis is staggering, as over 600,000 TKAs and 100,000 anterior cruciate ligament (ACL) reconstructions are performed annually in the United States [5, 6]. Incidence of arthrofibrosis after TKA varies, but can range between 1-13% [3, 7]. Arthrofibrosis is the most common complication of ACL reconstruction with reported rates between 4 and 38% [8–10].

Aside from surgery, joint trauma due to occupational or recreational injury can lead to arthrofibrosis [11]. Management of arthrofibrosis after trauma and surgery is poorly defined and ranges from physical therapy, bracing, oral corticosteroids, injectable corticosteroids and arthrolysis surgery with varying results [12]. To date, there have been no consistently successful therapeutics to combat arthrofibrosis following traumatic injury or surgical intervention [13].

**Fig. 1:**
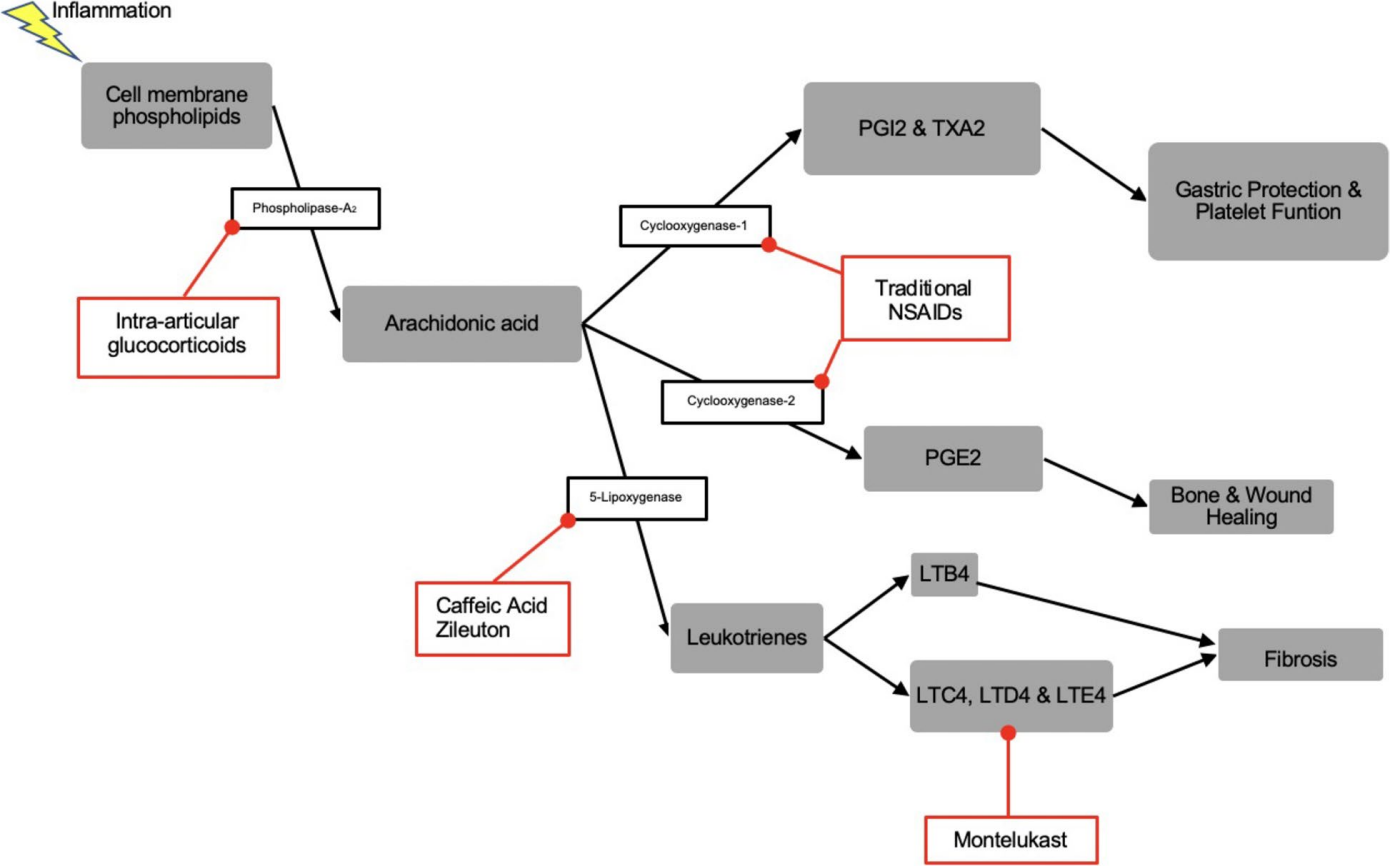
The eicosanoid pathway including relative metabolites and effects. Highlighted in red are the various treatment modalities that inhibit parts of this pathway. Metabolites include prostaglandin I_2_ (PGI2), thromboxane A_2_ (TXA2), and leukotrienes B_4_, C_4_, D_4_, and E_4_ (LTB4, C4, D4 and E4)

The pathogenesis of fibrosis is intimately associated with the inflammatory cascade and leukotriene activity [14]. Summarized in Fig. 1, Leukotrienes stimulate increased levels of inflammatory cytokines and are classified as cysteinyl (C4 and D4) or non-cysteinyl (B4) [15]. These inflammatory mediators lead to myofibroblast proliferation at the site of injury resulting in fibrosis [16, 17]. In joints, this fibrotic tissue can lead to painful capsular contracture. Previous investigations have demonstrated, in a joint contracture model, that intra-articular glucocorticoids, which inhibit prostaglandin and leukotriene activity, can strongly inhibit arthrofibrosis [18]. However, this therapy may also inhibit healing of bone and soft tissues through prostaglandin inhibition, as well as lead to an increased risk of infection [19, 20]. Previous work has shown cysteinyl leukotriene receptor inhibitors to have a moderate effect in preventing joint stiffness while these same compounds have been shown to accelerate fracture healing [21]. The ability of both cysteinyl leukotriene receptor inhibitors and intra-articular glucocorticoids to reduce joint stiffness suggests that leukotriene synthesis could play an important role in post-surgical fibrosis and stiffness.

5-lipoxygenase (5-LO) is the key enzyme in leukotriene production. 5-LO inhibitors have been shown to decrease the production of both cysteinyl and non-cysteinyl leukotrienes [22]. Currently, Zileuton is the only FDA approved 5-LO inhibitor, however, there are a number of organic compounds that have demonstrated 5-LO inhibitory effects, such as caffeic acid (CA) [23]. To our knowledge, no studies have explored using a 5-LO inhibitor, which would inhibit both cysteinyl and Leukotriene B4 (LTB4) activity, to prevent arthrofibrosis. In addition, no studies have clearly characterized that increased leukotriene activity is present with the post-traumatic joint contracture models.

This study investigated the hypothesis that leukotriene activity is increased in the post-traumatic knee joint, and that inhibition of leukotriene activity may prevent joint contracture after joint injury. The primary aim of our study was to evaluate the ability of a 5-LO inhibitor (CA) to prevent joint contracture in a surgical injury model (including bone defect) with post-surgical joint immobilization. Our secondary aim was to investigate if leukotriene activity is increased in our post-traumatic knee contracture model compared to the normal knee.

## Methods

This study was conducted from September 2017 to March of 2019. We used an animal model and obtained IACUC approval for use of live vertebrate animals (IACUS ID: 18–106.0-C.).

**Fig. 2:**
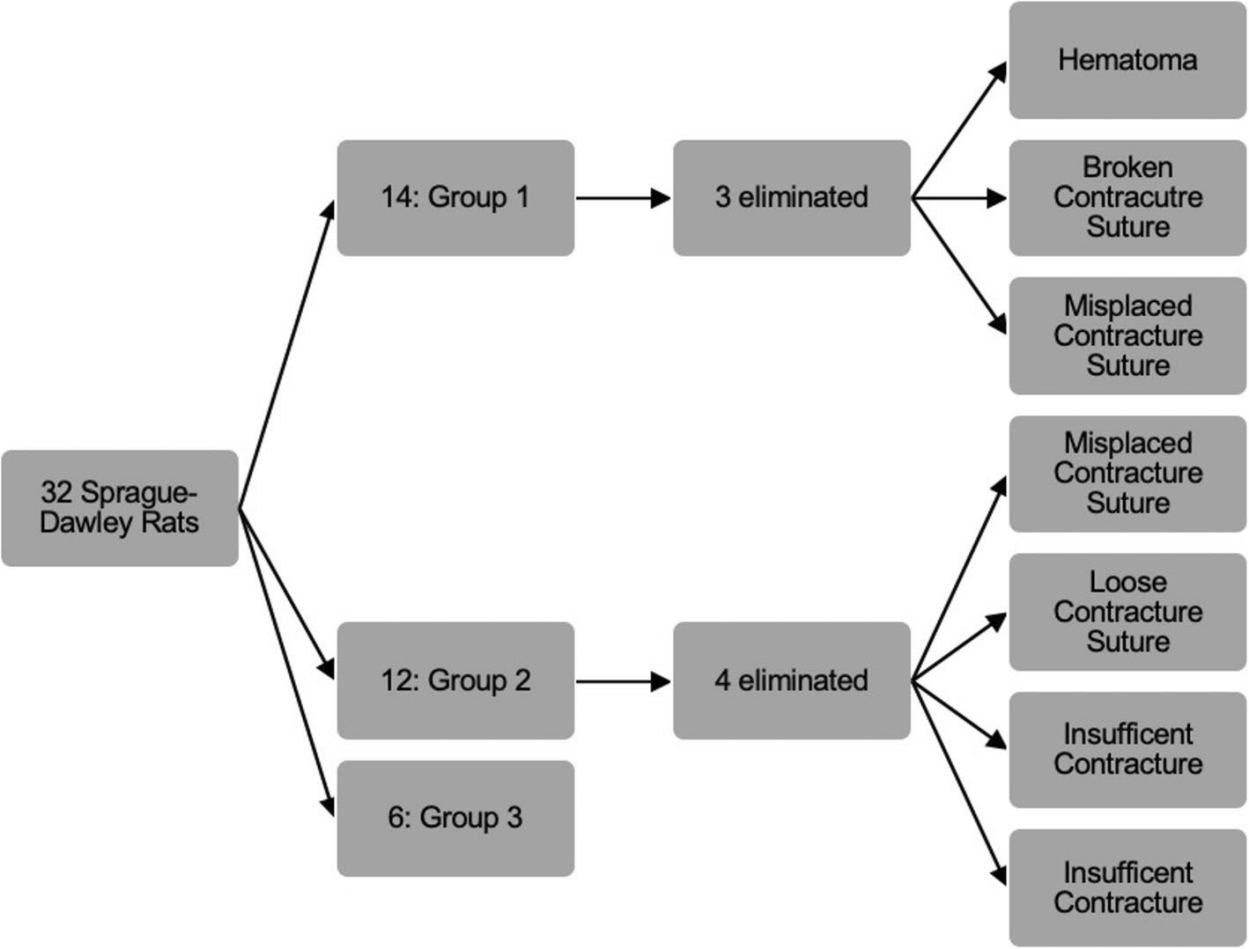
Male rats were obtained from the commercial vendor Charles River. Group 1: Surgical with CA in 10% ethanol (EtOH). Group 2: Surgical without CA in 10% EtOH. Group 3: Non-surgical controls. Group 2 received 10% EtOH on their food. Animals were assigned to groups based on weight. Average weight across all three groups was 488.53 g. Of the 32 animals, seven were eliminated for the above reasons

**Fig. 3:**
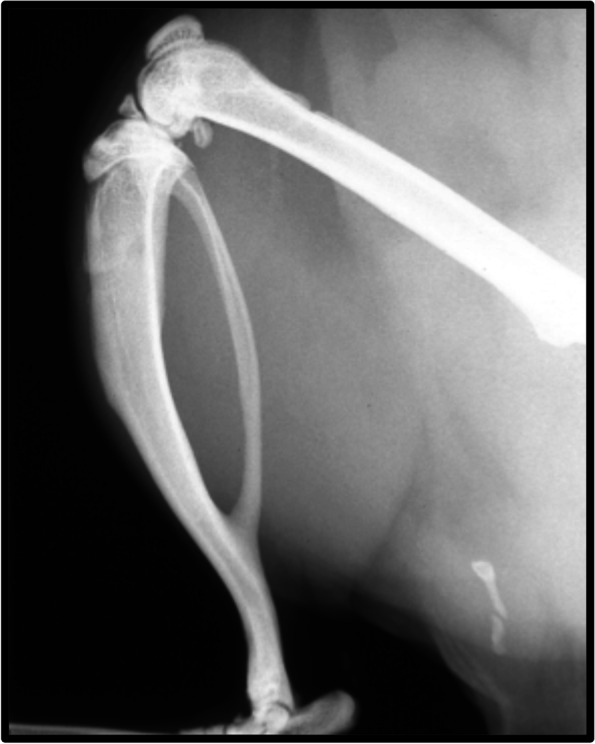
Radiograph of operative leg of a rat in the surgical without CA group with the immobilization suture intact

**Fig. 4:**
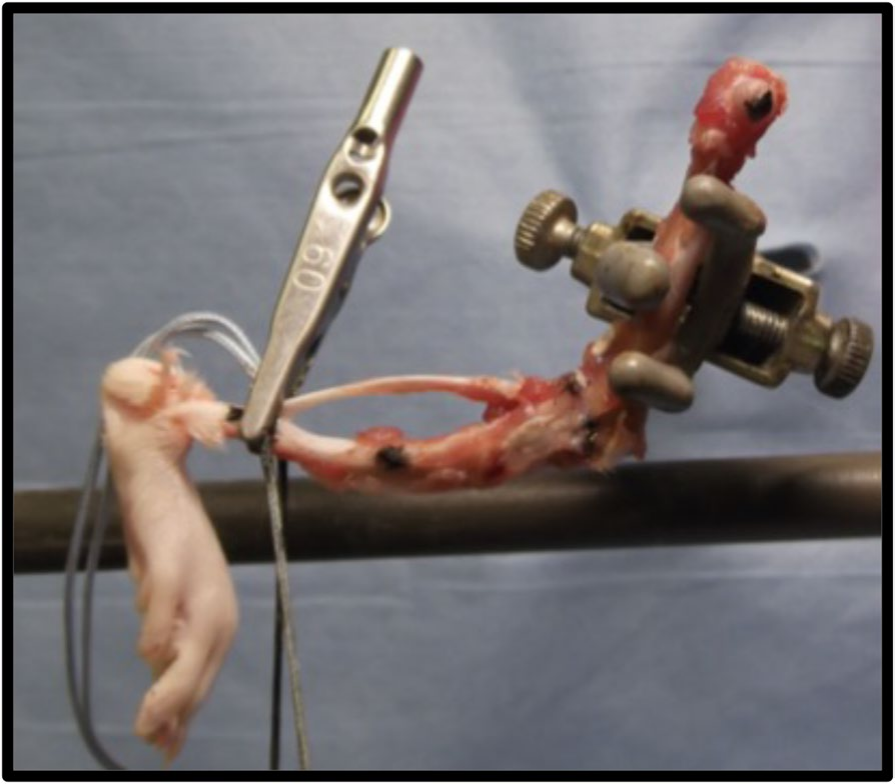
Apparatus used for FTA measurement in all operative and nonoperative limbs. Landmarks were designated with india ink at the medial malleolus, the origin of the MCL and the center of the femoral head

Under sterile conditions 32 rats were anesthetized with inhalational isoflurane (Fig. 2). Based on a surgical contracture model developed by Wilson et al., skin incision was made utilizing the medial parapatellar approach of the knee joint of the right side limb [24]. Following the knee arthrotomy, the patella was dislocated laterally and damage to the joint capsule and synovium was induced with a scalpel to simulate surgical and traumatic injury. An intraarticular fracture was created by drilling a 1.5–2.0 mm diameter cylindrical defect in the femoral intercondylar notch at the anterior aspect of the anterior cruciate ligament attachment. The joint capsule was closed with a 4–0 vicryl suture and the skin was then closed with wound clips. To immobilize the knees a femorotibial suturing procedure was performed to prevent motion and potentiate contracture and adhesion formation. A #2 Ethibond suture was passed extraperiosteally around the mid-portion of the femur and the tibia and tied with the knee flexed at approximately 125 degrees (Fig. 3). Prior to each surgery, acetaminophen (250 mg/kg) was present in the water of all animals for three days so that they were able to adjust to the taste of the elixir and subcutaneous buprenorphine was provided at induction. Postoperative analgesics included an acetaminophen elixir for 7 days postoperatively and subcutaneous buprenorphine 4–6 h after surgery and every 8–12 h for 48 h following surgery. Of the 26 rats, 12 were used in a surgical control group, designated surgical without CA, in which contracture was introduced and no CA treatment was administered. The remaining 14 rats were part of the surgical treatment group, designated surgical with CA, and received CA on their food daily. The surgical with CA group received food with CA 40 mg/kg, a dose tolerated in prior rat models [25]. Preliminary data showed that the average animal consumed only 20 pellets of food daily and thus were receiving less than the targeted dose of 40 mg/kg of CA. After this was recognized, each group was supplied with 25 new pellets daily and the consumption was tracked. The surgical with CA groups food was treated with the desired dosage of drug prior to administration each morning. The CA was suspended in 10% ethanol and then pipetted evenly over the 25 pellets; a technique adopted from the work of Tordoff et al. [26]. An additional 6 animals served as non-surgical controls and had no surgery performed on either limb but underwent the same feeding protocol as the surgical without CA group. The rats of the two surgical groups and the non-surgical control group were monitored daily. All animals from each group were housed individually so that food intake could be monitored. Wound clips were removed at 14 days. At 21 days after surgery the rats were euthanized. Immediately after euthanasia, blood was collected from the left ventricle of the heart for evaluation of plasma leukotriene levels. Subsequently, both hind limbs of all animals were disarticulated at the hip. All soft tissue was removed from the leg except for the joint capsule and collateral ligaments. For each animal, both limbs underwent femorotibial angle (FTA) measurements, which were recorded. Each limb was placed in a vice with a 0.015 Nm extension moment applied to the knee. The posterior knee was positioned upward, and the tibia was positioned parallel to the ground. A 41.76-g weight was hung 36.5 mm from the rotational center (origin of the MCL) of the knee. The FTAs were then measured with the joint capsule, collateral ligaments, cruciate ligaments and adhesions as the only structures limiting extension. Pictures were taken for each limb (Fig. 4). In a blinded fashion, the FTAs were measured digitally. The medial malleolus, the origin of the medial collateral ligament and the femoral head were used as landmarks for the measurements. For standardization in the surgical animals, the FTA for the nonoperative limb was subtracted from that of the operative limb to control for natural differences between the rats.

Subsequently, knees were processed for histology and sagittal sections were immunohistochemically stained for cells expressing 5-LO activity. Leukotriene levels of LTB4 were measured in the plasma and the patellar tendon/fat pad complex of the knee by immunoassay. Fracture healing was assessed with micro-computed tomography (micro-CT).

The rats were weighed at Day 0, 7 days, 14 days and at euthanasia (day 21). In addition, cage activity was logged as an indicator of distress. 7 days prior to the intervention 2 animals were used to determine daily food consumption which was used to ensure adequate food and drug delivery.

Consumption data was compared within and between groups. This data was used, with the animal weights, to assess adequacy of CA delivery to the treatment group.

A joint contracture angle was calculated as the difference in angle between operative and non-operative limb**.**

To assess systemic inflammation, plasma leukotriene levels were measured at the time of death.

To assess local inflammation, the patellar tendon with fat pad was isolated and leukotriene levels were compared to the non-operative limb of the same animal, the operative limbs of the treated animals and the limbs of the control animals. The leukotriene levels were evaluated with a commercially available R&D systems horseradish peroxidase enzyme-linked immunoassay kit.

**Fig. 5:**
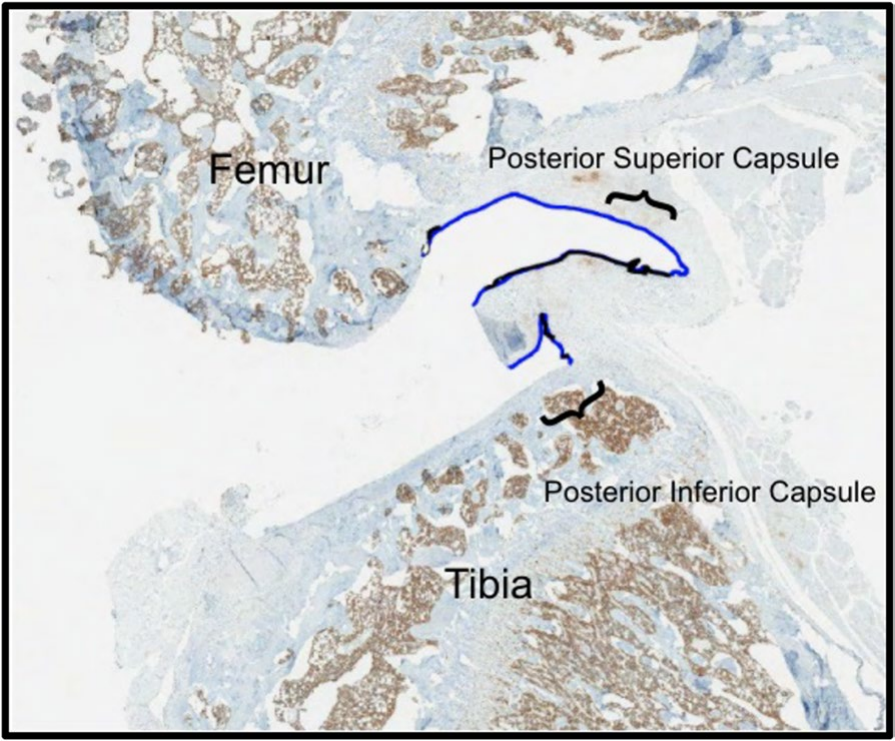
4 × 5 μm sagittal section of operative rat knee stained with Abcam anti-rabbit 5-LO antibody. Femoral and tibial bone marrow served as positive control for staining. Posterior capsule outlined in blue and posterior capsule demonstrating staining outlined in black

Histology specimens from half of the limbs (*N*=6/group) were fixed in 10% neutral buffered formalin, decalcified, and cut into 5 μm sagittal sections. Half of the slides were stained with safranin-O and the remaining unstained sagittal sections of the medial mid condylar region were used for immunohistochemistry to evaluate 5-LO positive cells in the posterior capsule per high power field. The unstained sections were stained with an Abcam anti-rabbit 5-LO antibody. Bone marrow served as a positive control for comparison. To quantify 5-LO activity in the posterior capsule, the percentage length of the posterior capsule demonstrating cells expressing 5-LO activity was measured by histomorphometry using the Aperio eslide manager with the embedded analysis software. To accomplish this, we measured the length of the posterior capsule, specifically the synovial intima from the proximal posterior superior horn of the medial meniscus to the distal posterior inferior horn of the medial meniscus. We then measured the length of the posterior capsule demonstrating 5-LO activity by identifying areas with staining, as shown in Fig. 5. For each limb, two sections were assessed, and the median value was reported over the interquartile range. Differences in contracture angles, posterior capsule length and leukotrienes between the groups were evaluated by a paired Student T-test and unpaired Mann-Whitney rank sum test.

## Results

In the first week following surgery, we anticipated a modest 5–10% loss in body weight due to a decrease in PO intake, as is common following surgery with anesthesia and the pica response to buprenorphine administration. There was weight loss among the surgical animals, but it was modest and equal among groups as can be seen in Table 1.

**Table 1:** Mean weights from time of surgery to time of time (T) = 0 Days to T = 21 Days

Group	Time of Surgery Mean Weight (g)	1 Week Post-Op Mean Weight (g)	2 Week Post-Op Mean Weight (g)	Time of Death Mean Weight (g)	Mean Percent Weight Loss (g)
Surgical with CA	498 ± 19.1	463 ± 14.8	485 ± 17.9	493.9 ± 17.8	0.813 ± 1.49
Surgical without CA	507.3 ± 24.7	472 ± 22.9	494 ± 26.6	502 ± 24.9	1.04 ± 1.04

**Fig. 6:**
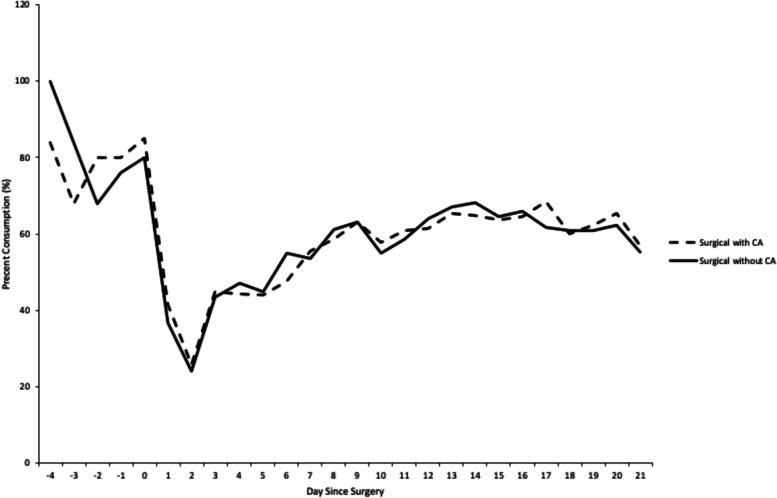
Daily food consumption was recorded and converted into a percent consumption. Pre-surgery data indicated a mean 80% of total daily consumption

**Fig. 7:**
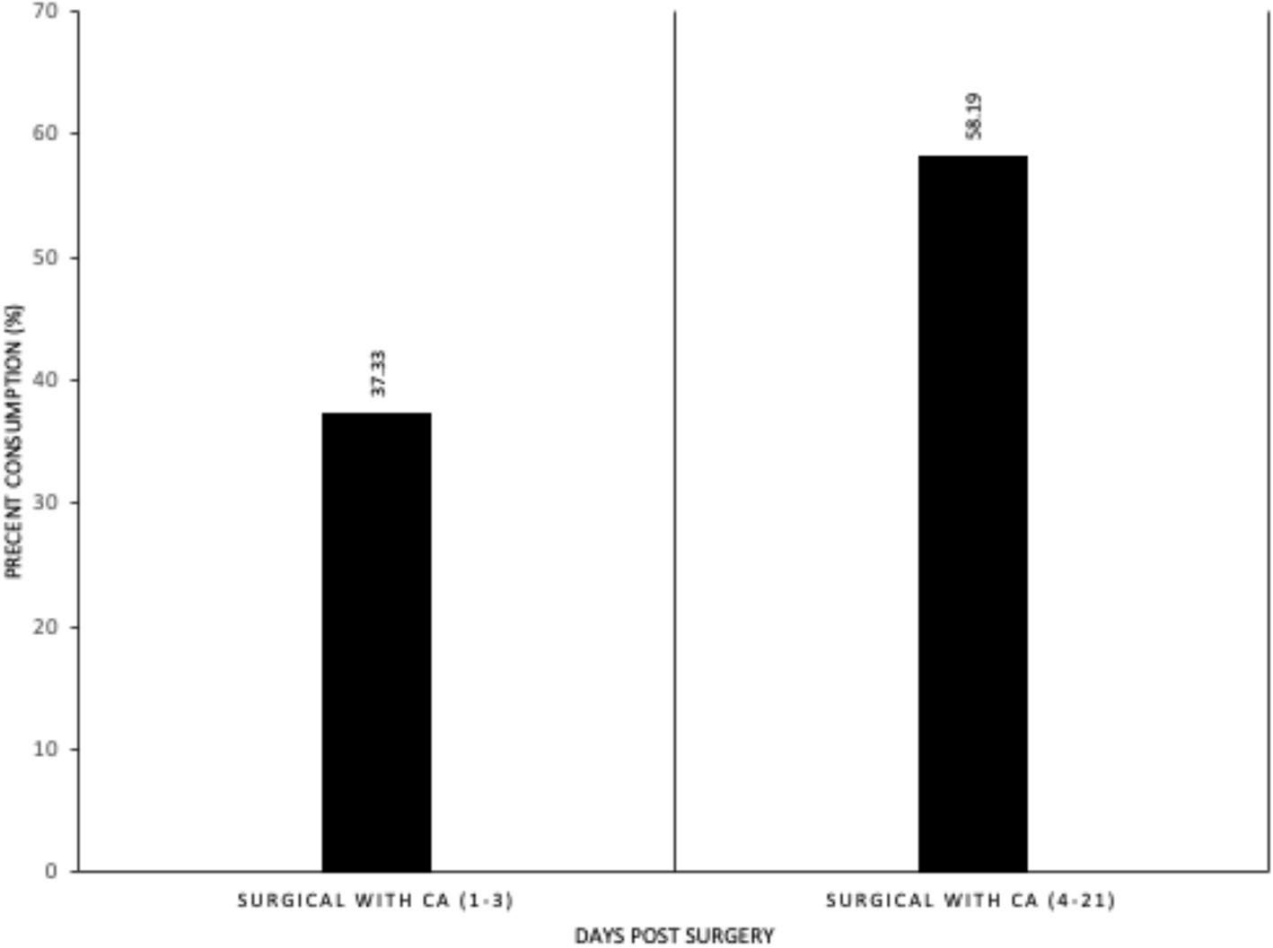
Food consumption in the caffeic acid treatment group during postoperative days 1–3, when buprenorphine was dosed, compared to days 4–21 when no buprenorphine was used

In the pre-operative period, our pilot animals consumed a 55 mg/kg/day diet as expected**.** However, Figs. 6 and 7 show that postoperative consumption decreased and was more pronounced in the immediate (days 1-3) postoperative period. Food consumption greater than 80% was needed to deliver the desired 40 mg/kg of CA as demonstrated in previous animal models [25].

As shown in Table 2, The surgical without CA group and surgical with CA group developed a 32˚ and 29˚ loss of extension as compared to the contralateral non-immobilized knee, respectively. Joint contracture angles were not statistically different between the surgical with CA and surgical without CA group.

**Table 2:** Mean suture intact angle and FTA-intact knee angle, by group

Group	Mean Suture Intact Angle (˚)	Mean FTA-Knee Intact Angle (˚)
Surgical with CA	124.4 ± 6.7	29.8 ± 4.3
Surgical without CA	126.4 ± 6.9	32.0 ± 6.6
*P*-Value	0.55	0.4

As shown in Tables 3 and 4, Plasma LTB4 levels were not significantly different between the surgical with CA and the surgical without CA group. Similarly, PT/FP LTB4 levels were not significantly different between the surgical with CA and the surgical without CA group. LTB4 levels were found to be significantly lower in the non-surgical control animals when surgical with CA and surgical without CA animals were grouped together and compared with a Mann Whitney rank sum test.

**Table 3:** Mean Leukotriene B4 (LTB4) concentration in the blood plasma at the time of death

Group	Mean LTB4 Concentration—Plasma (ng/mL)
Surgical with CA	36.6 ± 14.4
Surgical without CA	33.7 ± 11.4

**Table 4:** Mean LTB4 Concentration in the Patellar Tendon (PT) and Fat Pad (FP)

Group	Mean LTB4 Concentration—PT/FP (pg/mL)
Surgical with CA	165.1 ± 55.6
Surgical without CA	147.1 ± 59.5
Surgical with CA + Surgical without CA	*157.6 ± 55.3
Non-surgical Control	107.79 ± 34.08

^*^Significantly different from control (*p* < 0.05)

**Fig. 8:**
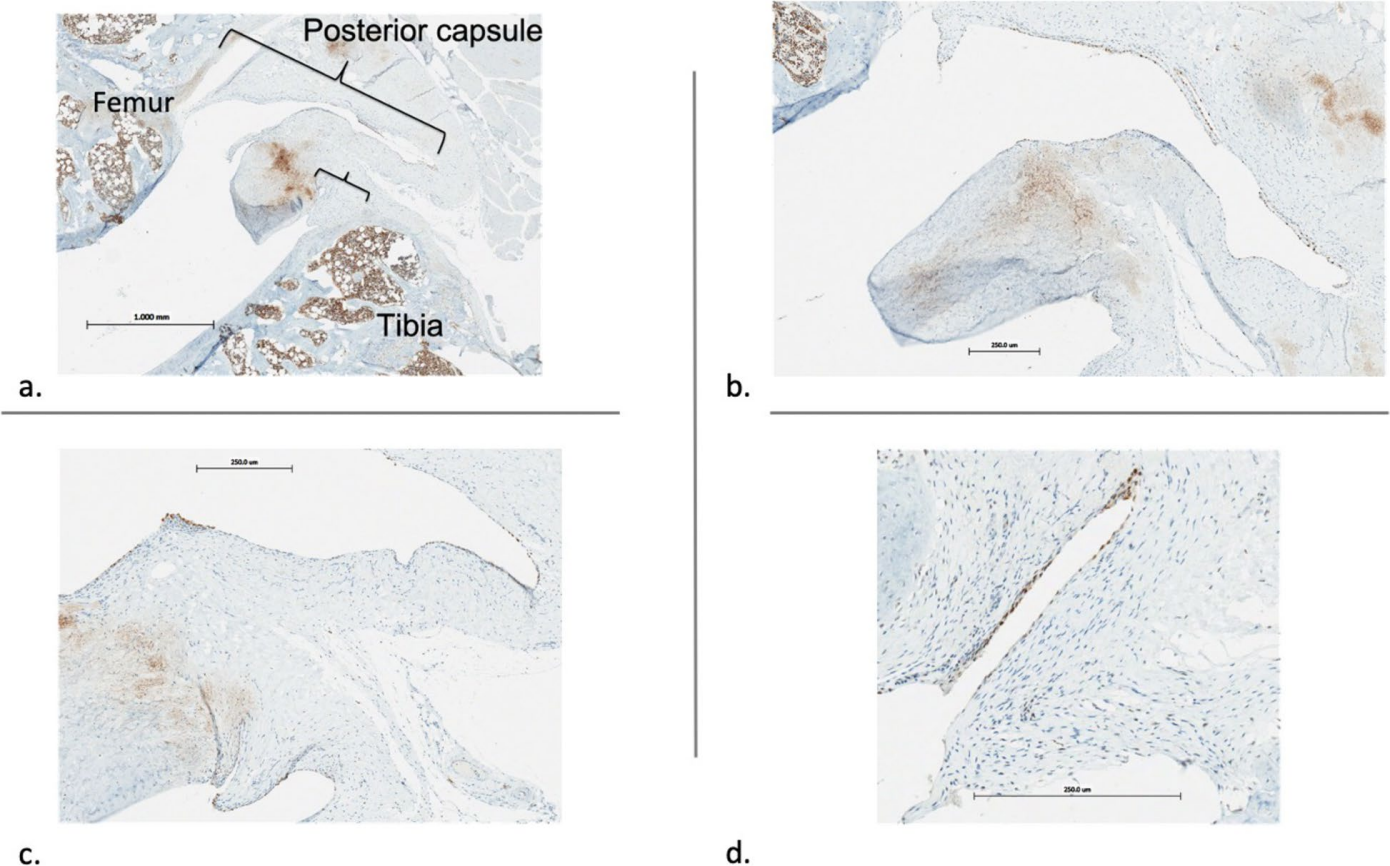
Immunostaining for 5-LO done on 5 μm sagittal sections of rat knee joint with the posterior capsule intact. Sections are from the medial mid-condylar region. **a** 1 × image of medial femoral condyle, medial tibial plateau and associated posterior capsule of an immobilized animal. Positive 5-LO activity is represented by uptake of brown stain. Bone marrow in femur and tiba represent a good internal control. **b**, **c** and **d** are 4 × images of (a) highlighting the staining in the posterior capsule of an immobilized animal

The percentage length of the PC with 5-LO activity was found by averaging the portion of the PC demonstrating 5-LO staining (Fig. 8). Reported as the median over the IQR. Based on this analysis, cells expressing 5-LO activity were significantly increased in the combined surgical with CA and surgical without CA group compared to the naive control animals when compared with a Mann Whitney rank sum test.

## Discussion

Given the loss of extension in both the surgical with CA and surgical without CA groups, we successfully induced contracture in all surgical animals compared to contralateral limbs. Surgical immobilization in the surgical with CA and surgical without CA groups, when combined, resulted in statistically significant increased 5-LO activity of the synovial surface of the posterior joint capsule, compared to the non-surgical control. Previous work in our lab and the literature to date have demonstrated increased alpha smooth muscle actin, transformation growth factor beta 1, hyaluronic acid and type A/B synoviocytes, but not 5-LO activity [27]. In addition, we showed statistically significant increases in LTB4 levels in the patellar tendon and fat pad after trauma/simulated surgical intervention in the combined surgical with CA and surgical without CA groups, compared to the nonsurgical control. In our model, the percentage of the posterior capsule length demonstrating 5-LO activity correlated with the degree of contracture. Immunostaining of the posterior capsule based on prior method by Ando et al showed that 5LO is elevated at the site of this remodeling [27]. This substantiates our conclusion that 5-LO and its products LTB4 and cysteinyl leukotrienes (cysLTs) contribute to the inflammatory response during knee joint contracture. This is similar to prior reported effects of the 5-LO inhibitor, CA, as anti-inflammatory agent in cardiac tissue and wound healing [28–30]**.** As well as, the fibrogenic nature of leukotrienes previously documented in fibrotic lung pathologies, such as idiopathic pulmonary fibrosis and bleomycin induced fibrosis [31].

However, we were unable to reduce the degree of contracture with 5-LO inhibition using CA compared to the surgical without CA group. In addition, oral administration of CA alone was ineffective at reducing plasma LTB4 levels, patellar tendon—fat pad LTB4 levels, or PC 5-LO activity, compared to the surgical without CA group.

These findings represent that optimal dosing of CA is likely required to reduce the degree of joint contracture, levels of plasma LTB4 and PC 5-LO activity and thus was not seen in our model due to suboptimal dosing. This is within reason given our difficulty in achieving adequate oral CA intake in the immediate postoperative period. As shown in Fig. 1, in the immediate postoperative period (days 1–3) daily food consumption dropped from a baseline of 80% to 37.3%. The percent consumption rebounded after buprenorphine analgesia was discontinued on day 4; increasing to a mean of 58.2% for days 4 to 21 [32]. However, the immediate postoperative period has been shown to be a time of significant tissue remodeling and inflammation [33]. Therefore, the suppressed food intake in the immediate postoperative period likely led to limited CA at the site of injury during the period of significant inflammation and no 5LO inhibitory effect. Therefore, though unsuccessful in this experiment, inhibiting 5-LO activity may still be effective in preventing arthrofibrotic changes.

Oral delivery of CA has been well cited in the literature [28–30, 34]. CA has limited first pass metabolism and good gastrointestinal absorption [34, 35]. It is difficult to solubilize though and required a solution of at least 10% ethanol to prevent precipitation. We considered subcutaneous administration of CA to ensure a consistent daily dose but were limited by its solubility in water. For the dosage we required a solution of 5 mL would have been needed to be injected daily. This was an excessive volume and the percentage of ethanol in our smaller volume preparation was not feasible for injection either. Rats are known to tolerate ethanol orally and in our preliminary consumption data supported this [26]. The limiting factor in our preparation seems to have been the application of the CA solution daily to the food. For future studies, the preparation should be applied to a smaller portion of food, lavaged, or delivered in microspheres at the time of surgery to the site of injury.

Proper delivery of a 5-lipoxygenase inhibiting drug, such as CA, has potential to decrease posterior synovial capsule 5-LO activity, leading to lower LTB4 and cysLT levels with a subsequent reduction of joint fibrosis and joint contracture, as shown in other models of fibrosis [28–30]. Elevated LTB4 in the patellar tendon—fat pad and increased 5-LO staining in the posterior capsule in our joint contracture model support that 5-LO is a reasonable target for inhibition of the tissue remodeling seen with post traumatic joint contracture.

## Conclusions

Surgical immobilization resulted in increased 5-LO activity of the synovial surface of the posterior knee joint capsule and increased LTB4 levels in the patellar tendon - fat pad in our animal model. Oral administration of the 5-LO inhibitor CA was ineffective at reducing systemic (plasma) LTB4 levels, local (patellar tendon - fat pad) LTB4 levels and preventing knee joint contracture in this study, but likely due to suboptimal dosing of CA. Inhibiting 5-LO activity, though unsuccessful in this experiment, may be effective in preventing arthrofibrotic changes if adequate dosing can be achieved and should be explored in future studies.

## Data Availability

The datasets used and analyzed during the current study are available from the corresponding author on reasonable request.

## Abbreviations

CA: Caffeic acid
5-LO: 5-Lipoxyoxygenase
EtOH: Ethanol
PT: Patellar tendon
FP: Fat pad
cysLT: Cys-leukotriene
PGI2: Prostoglandin I2
TXA2: Thromboxane 2
LTB4: Leukotriene B4
